# Identification of *Lama glama* as Reservoirs for *Acinetobacter lwoffii*

**DOI:** 10.3389/fmicb.2017.00278

**Published:** 2017-03-02

**Authors:** Martín M. Ledesma, Ailén M. Díaz, Claudia Barberis, Carlos Vay, Marcela A. Manghi, Juliana Leoni, Marisa S. Castro, Alejandro Ferrari

**Affiliations:** ^1^Facultad de Farmacia y Bioquímica, Instituto de Estudios de la Inmunidad Humoral (CONICET), Universidad de Buenos AiresBuenos Aires, Argentina; ^2^Laboratorio de Análisis Clínicos, Hospital de Clínicas José de San MartínBuenos Aires, Argentina

**Keywords:** *Lama glama*, *Acinetobacter*, serology, MALDI/TOF-MS

## Abstract

South American Camelids have an increasing relevance in local economies, worldwide. These animals are bred for their meat, fur and as companion and therapy animals. Thus, their sanitary status should be well-established. According to the OIE (World Organization for Animal Health), respiratory infections mainly produced by *Pasteurella* spp. have been reported for camelids. It has been stated that this microorganism causes a mild disease, although many authors report it is an important cause of mortality among alpacas. Nevertheless, the incidence of infection by *Pasteurella* spp. in camelids still needs to be investigated. The aim of the present study was to analyze the occurrence of nasopharyngeal colonization of *Lama glama* by respiratory bacteria, and to assess the usefulness of serological tests for clinical diagnosis. The colonization was studied by culture techniques carried out with material taken by nasopharyngeal swabs. Bacterial isolates were first phenotypically characterized and then identified by MALDI/TOF-MS. The presence of specific serum antibodies was studied by ELISA and Western blot. In the present work *Pasteurella* spp. was not found. Nevertheless, we report for the first time, the colonization of *L. glama* by bacteria of the *Acinetobacter lwoffii*, at a reliable level in 19.4% of the animals. *Acinetobacter* species are found in different environmental sources, as well as vegetables, animals, and humans, and their role in infections has recently gained relevance. The results presented herein contribute to a better understanding of the respiratory microbiota in camelids, and increase the knowledge about environmental distribution of *Acinetobacter* non-*baumanii* species. Given that these respiratory bacteria might be the cause of infection among cattle, and even humans, this report highlights the need for further research.

## Introduction

The family of South American camelids (SACs) comprises four species: llama (*Lama glama*), alpaca (*Lama pacos*), guanaco (*Lama guanicoe*), and vicugna (*Vicugna vicugna*). These animals are bred for their meat, fur and as companion and therapy animals, which gives them an increasing relevance worldwide. Thus, it is clear that their sanitary status should be studied in depth, including the assessment of SACs as potential transmitters of diseases to other animals or even to humans.

Considering the close contact that SACs might have with humans, and taking into account that these are commonly bred together with bovine, equine, ovine, and porcine cattle, diseases transmitted by fluids and feces are to be controlled. The occurrence of respiratory opportunistic pathogens in South American camelids (SACs) has been reported, and therefore it should be monitored, as by serological tests. However, many studies have indicated that the use of serological tests in camelids deserve further analysis. For example, a global study of infectious diseases conducted in the *Camelidae* family during the 80s indicated that the most frequent opportunistic bacterium associated with respiratory diseases was *Pasteurella multocida* (Fassi-Fehri, 1987). In that study, authors found 47% of carriers and 80% of seropositive animals for *P. multocida* within Old World camelids (camels and dromedaries), thus indicating a lack of correlation between direct analysis and serology.

During 2008, the OIE published a report on “Diseases of Camelids.” In this report a classification was proposed, grouping significant diseases (either viral, bacterial or parasitic) of SACs and Old World camelids into three categories, depending on their prevalence and the knowledge about their pathogenicity. In that list, *Pasteurella* was the only bacterial agent reported in SACs as well as in cattle. Pasteurelosis was classified within group III (mild disease), for which SACs had unknown susceptibility. Besides, no serological tests were available to detect this pathogen (OIE, 2008). Although the 2010 OIE report associated *Pasteurella* with hemorrhagic septicemia, some authors reported an outbreak of *P. multocida* and *Mannheimia haemolytica* in Peru, which resulted in acute pneumonia (Rosadio et al., 2011; Guzmán et al., 2013). These reports provide substantial information to affirm that pasteurelosis may be a much more serious disease than the OIE reports suggest.

Considering the context, the present study was aimed at analyzing the occurrence of nasopharyngeal colonization of *L. glama* by respiratory bacteria belonging to the genres *Acinetobacter, Pasteurella*, and *Mannheimia*, and to assess the value of serology for clinical diagnosis. The assessment of colonization was conducted by the analysis of material taken by nasopharyngeal swabs. Colonies were then identified by MALDI/TOF-MS. The serology was performed by both enzyme-linked immunoanassay (ELISA) and Western blot, using a protein extract obtained from cultured isolates. ELISA assays were designed to analyze the existence of a correlation between the presence of specific antibodies to the whole protein extract and direct culture of nasopharingeal swabs, and Western blot assays were performed to evaluate wether that correlation occurred only with some proteins from the extract. In order to evaluate its performance and usefulness, the serological tests employed herein were then evaluated employing a larger set of samples obtained in several regions of Argentina.

## Materials and methods

### Animals and samples

The study was conducted with 36 healthy llamas (*L. glama*) from La Hoyada, Santa María, Catamarca, Argentina, ranging from 1 to 3 years old. Animals were kept in a herd of more than 200 individuals, grazing along with bovine and ovine cattle. No history of respiratory disease or unexpected deaths had been previously reported for this herd, neither during nor after the protocol was conducted. Nasopharyngeal samples were taken—one from each animal—in sterile transport swabs with Amies medium (Copan Italia, Brescia, Italy) and stored at 4°C until microbiological analysis. All blood samples were collected by femoral vein puncture. Blood samples were allowed to clot at room temperature, and sera were separated by centrifugation, aliquoted and stored at −20°C until used.

A further serological analysis was conducted using samples from a previously established serum bank, containing a collection of serum samples of *L. glama* from different regions within Argentina (provinces of Buenos Aires, San Luis, Chubut, Jujuy, and Entre Ríos). The bank also included 14 serum samples from *L. guanicoe*. All these samples were collected by jugular vein or femoral artery puncture during a long-term campaign conducted from 2004 to 2014. Serum samples were stored at −20°C until use, and analyzed by the serological assays described in the corresponding section of this work.

### Microbiological culture and analysis

Nasopharyngeal samples were plated on brain-heart agar (BHA) and blood agar (Britania laboratories, Buenos Aires, Argentina). Colonies were selected based on their morphological characteristics, selecting the ones that showed high similarity with *Acinetobacter/Pasteurella*/*Mannheimia* colonies (small, transparent and well-defined colonies). All isolates were sub-cultured from stock cultures in 5% rabbit blood agar and incubated under aerobic conditions at 37°C for 24 h. Briefly, the biochemical characteristics of each colony were assessed by indole production, catalase reaction, Gram staining, sensitivity to penicillin (10 IU/ml) and streptomycin (10 μg/ml) (Gibco, Thermo Fisher Scientific, New York, USA), and their development in chromogenic agar (Chromobrit IU, Britania laboratories, Buenos Aires, Argentina), comparing them with known positive cultures (phenotypic profile identification and 16S rRNA sequencing, Macrogen Inc., Korea). Biochemical reactions were observed after inoculation of 1–2 colonies of pure culture.

Colonies matching biochemical characteristics of *Acinetobacter/ Pasteurella*/ *Mannheimia* were selected for further identification. Colonies were selected if positive for the following criteria: indole reaction, catalase reaction, Gram staining (negative *bacillococci*), sensitivity for both antibiotics and positivity for the development in chromogenic agar. A sample known to be *P. multocida* was processed as a positive control, and an isolate coming from the apex of the lung was used as a negative control, given that it showed growth in the shape of brown colonies (hence, not compatible with none of the microorganisms analyzed).

Microbial identification was performed by means of matrix-assisted laser desorption ionization–time of flight mass spectrometry (MALDI/TOF-MS). Briefly, mass spectra were acquired using the MALDI-TOF MS spectrometer in a linear positive mode (Microflex, Bruker Daltonics, Massachusetts, USA). The bacterial test standard (Bruker Daltonics, Massachusetts, USA) was used for instrument calibration. Bacterial identification was performed using the corresponding library and software (MALDI Biotyper library 3.0 and MALDI Biotyper software version 3.1, Bruker Daltonics, Massachusetts, USA). As indicated by the manufacturer and reported elsewhere (Alatoom et al., 2012), cut-off scores for identification were: scores ranging from 2.00 to 3.00, considered “high confidence identification;” scores ranging from 1.70 to 1.99, considered “low confidence identification;” and scores lower than 1.7, considered “not reliable.”

### Antigen preparation for immunoassays

Antigens for immunoassays were prepared from isolated and identified colonies. The corresponding bacteria were grown in tryptone soy broth (TBS) at 37°C with continuous shaking. The culture medium was then centrifuged at 4,000 rpm for 15 min at 4°C. The pellet was then washed three times with phosphate-buffered saline (PBS), and bacterial lysis was achieved by suspending the pellet in 10 ml of cold PBS and performing three cycles of freezing in liquid nitrogen followed by thawing. Bacterial cells were then sonicated in 3 pulses of 15 s (Elma Transsonic 540 Sonicator, Singen, Germany), centrifuged at 10,000 rpm for 30 min at 4°C, and the supernatant separated for further use. The total protein content was assessed by the BCA method (Pierce Biotechnology, Thermo Fisher Scientific, New York, USA), and the lysate was aliquoted and stored at −20°C until use as antigen for serological analyses.

### Detection of specific antibodies by enzyme immunosorbent assay (ELISA)

Specific antibodies in individual serum samples were detected by indirect ELISA, using the antigen preparation described above, as coating antigen. Briefly, polystyrene microtiter plates (Nunc MaxiSorp, Rochester, New York) were coated with 1 μg/well of protein extract, during 1 h at 37°C. Afterwards, plates were washed with PBS containing 0.05% Tween 20 (PBS-T), and then blocked with 300 μl of 3% non-fat dry milk in PBS (PBS-M) overnight at 4°C. Plates were then washed three times with PBS-T and incubated with each llama serum, diluted 1:100 in PBS-M for 1 h at 37°C. After washing three times, bound antibodies were detected by incubating with 100 μl of a HRP-labeled goat anti-llama serum (Bethyl, Montgomery, USA) diluted at 1:7,000 in PBS-M, during 1 h at 37°C. Wells were washed, and the reaction was developed with 100 μl of the substrate-chromogen (3,3′,5,5′ tetramethylbenzydine) (Thermo Fisher Scientific, New York, USA). The reaction was stopped by the addition of 50 μl 4 N H_2_SO_4_. Optical density (O.D.) values at 450 nm were determined in a microplate reader (Original Multiskan Ex, Thermo Electron Corporation, Thermo Fisher Scientific, New York, USA). Given that there are no commercial nor in-house control sera available, data analysis was conducted as stated in the Section Statistical Analysis.

### Detection of specific antibodies by western blot

Western blot assays were performed to evaluate wether there was a correlation between the antigen recognition pattern and direct culture of nasopharingeal swabs. Serum samples were chosen among those which provided high O.D. values by ELISA –to guarantee that any absence of signal was not due to lack of specific antibodies–, selecting some that resulted culture-positive and some culture-negative. Briefly, proteins were separated by 12% polyacrylamide gel electrophoresis (SDS-PAGE), loading up to 30 μg per lane. Proteins were then transferred to a PVDF (PVDF, Plus Transfer membrane, 0.22 Micron GE Water & Process Technologies, Little Chalfont, UK) membrane using an immunoblotting device (Bio Rad, California, USA). Membranes were then blocked with 3% w/v BSA in PBS-T (PBS-T-B) overnight at 4°C. Proteins were detected using llama sera diluted 1/100 in PBS-T-B for 1 h at room temperature. Specific antibodies were detected using a HRP-labeled goat anti-llama (H+L) conjugate, at a 1:7,000 dilution for 1 h at room temperature with continuous shaking. After each incubation step, membranes were washed with PBS-T twice. Phosphate and Tween were removed in a final step by washing membranes with Tris buffered saline (TBS). Membranes were then exposed to enhanced chemiluminescence commercial substrate (Pierce ECL WB Substrate, Thermo Fisher Scientific, New York, USA) and revealed in a photographic film (Kodak, Rochester, NY, USA).

### Statistical analysis

ELISA optical density values were analyzed by calculating the mean, median, standard deviation (*S.D*.) and distribution by the Shapiro-Wilks normality test. Animals were classified as positive or negative according to the microbiological status of each animal and also distinguished depending on the region of origin.

The lack of reference values made it necessary to develop a specific approach for the definition of positive serology. To achieve this, the presence of outlier values was detected in each group by means of a “robust test.” The number of outliers was registered, as well as the alteration of each group distribution before and after the elimination of outliers. The process was repeated until no sample could be eliminated, yielding either a normal or a non-normal set of values. Eliminated outliers were initially considered positive by the serological method, and their O.D. values were then compared with those that had not been eliminated (considered negative for the serological test), using the Mann-Whitney's *U*-test. A further comparison between values derived from animals belonging to different regions was conducted using the Kruskal-Wallis' test (non-parametric ANOVA), followed by Dunn's Multiple Comparisons Test.

## Results

### Microbiological analysis and identification

Bacterial swabs were successfully cultured from the 36 animals, and isolations were performed. However, 13 isolates did not have morphological compatibility with respiratory bacteria and were hence discarded. Two other isolates resulted in a very complex mixture of bacteria, and were then considered not suitable for our study. The remaining 21 isolates, as well as three additional isolates derived from lung parenchyma, apex and base, obtained from a euthanized animal (the three considered negative for *Pasteurella/Mannheimia* due to colony morphology, but included as internal negative controls) were subjected to further biochemical identification (Table 1). From these, only 14 colonies had biochemical properties compatible with *Acinetobacter/Pasteurella*/*Mannheimia*. Although one of the 14 proved to be *Arthrobacter oxydans* (with a score of 2.11) and 6 out of 14 had “not reliable” scores for *Acinetobacter lwoffii*, the remaining 7 were confirmed to be *A. lwoffii* with a reliable score (4 of them with scores ranging from 1.70 to 1.99, and 3 of them with scores ranging from 2.00 to 3.00), as seen in Table 1. Taken together, these results show that at least 19.4% of the animals (7 out of 36) carried *A. lwoffii*, as identified with a reliable score by the MALDI/TOF-MS method. This value could be used as an estimation of *A. lwoffii* colonization rate in the herd.

**Table 1:** **Microbiological analysis of isolates and bacterial identification by MALDI/TOF-MS**.

**Animal ID**	**Ampicilin/Streptomycin**	**Morphology, Gram stain**	**Catalase reaction**	**Chromogenic agar growth**	**Indole Reaction**	**MALDI/TOF-MS identification**
l302	Sensitive	Coccobacilli, G−	+	+++	+	*A. lwoffii (1.49)*
l304	Sensitive	Coccobacilli, G−	+	+++	+	*A. lwoffii (2.08)*
l307	Sensitive	Coccobacilli, G−	+	+++	++	*A. lwoffii (1.72)*
l308	Sensitive	Coccobacilli, G−	+	+/−	++	*A. lwoffii (1.67)*
l309	Sensitive	Coccobacilli, G−	+	+++	++	*A. lwoffii (1.51)*
l310	Sensitive	Coccobacilli, G−	+	+/−	+++	*A. lwoffii (1.77)*
l311	Sensitive	Coccobacilli, G−	+	++	+	*A. lwoffii (1.58)*
l312	Sensitive	Coccobacilli, G−	+	−	−	*N/A*
l313	Sensitive	Coccobacilli, G−	+	+/−	−	*N/A*
l314	Sensitive	Coccobacilli, G−	+	++	++	*A. lwoffii (1.76)*
l318	Sensitive	Coccobacilli, G−	+	++	++	*A. lwoffii (1.46)*
l320	Sensitive	Coccobacilli, G−	+	−	−	*N/A*
l321	Sensitive	Bacilli, G+	+	−	+++	*N/A*
l325	Sensitive	Coccobacilli, G−	+	−	+++	*A. lwoffii (1.5)*
l328	Sensitive	Coccobacilli, G−	+	−	−	*N/A*
l329	Sensitive	Coccobacilli, G−	+	+	+	*A. lwoffii (2.02)*
l333	Sensitive	Coccobacilli, G−	+	+++	+	*A. lwoffii (1.87)*
l334	Sensitive	Coccobacilli, G−	+	+	+	*Arthrobacter oxydans (2.11)*
l335a	Sensitive	Coccobacilli, G+	+	−	−	*N/A*
l335b	Sensitive	Coccobacilli, G−	+	−	−	*N/A*
l600	Sensitive	Coccobacilli, G−	+	+	+	*A. lwoffii (2.09)*
L.P.	Resistant	Coccobacilli, G−	+	+	+	*N/A*
Lung Apex	Sensitive	Bacilli, G+	+	−	+	*N/A*
Lung Base	Sensitive	Coccobacilli, G−	+	+	+	*N/A*

*Animal ID corresponds to the identification tag of each individual. L.P. stands for lung parenchyma, and Lung Apex and Lung Base are two isolates considered negative for Pasteurella/Mannheimia. Chromogenic agar growth was considered positive (+, ++, or + + + according to the aspect-size and color- of colonies), negative (−) or indefinite (+/−). Identification score yielded by the MALDI/TOF-MS analysis is indicated between brackets. N/A indicates the cases in which the MS analysis was not performed because of an incompatible biochemical profile*.

### Detection of specific antibodies by ELISA

**Figure 1:**
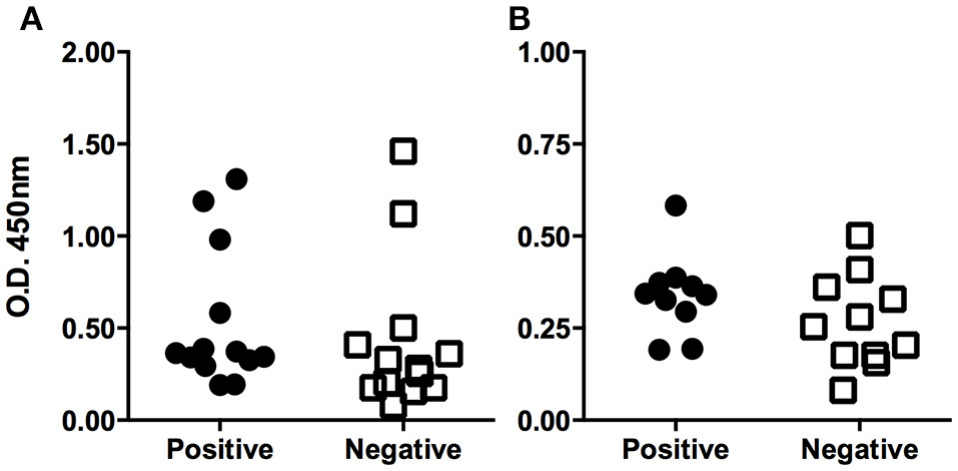
**Serological determination of specific antibodies against ***Acinetobacter lwoffii***. (A)** The figure shows the ELISA optical densities corresponding to each serum sample when tested against *Acinetobacter lwoffii* antigen preparation. Animals were grouped according to the result of direct culture of nasopharingeal swabs. Outliers can be identified by simple observation. Panel **(B)** shows the same data after discarding the outliers; the normal distribution can be observed.

According to the microbiological analysis, positive and negative populations were defined for the presence of *Acinetobacter* spp. To check if these populations differed in terms of the level of specific antibodies, an ELISA was used as described above. Normality of data was assessed by the Shapiro-Wilk test, resulting in two non-normal distributions (*P* < 0.1; Figure 1A). Therefore, the difference was studied by the Mann-Whitney non-parametric test, resulting in a *P*-value of 0.0928 (one-tailed test), which was considered non-significant. A robustness test for the presence of outliers was conducted, and outliers were found in both populations (Table 2). The elimination of these values eventually led to a normal distribution for both populations, although an unpaired Student's *t*-test still indicated the difference was not significant (the two-tailed *P*-value was 0.1692; Figure 1B).

**Table 2:** **Detection of outliers by the robust test**.

**Group (direct analysis result)**	***n***	**Mean O.D**.	***S.D***	**Mean**	**Shapiro-Wilks (*p*-value)**	**Number of outliers**
Positive	13	0.53	0.38	0.36	0.0010	3
Negative	13	0.42	0.41	0.28	0.0002	2

*Table indicates the number of outliers detected in each set of samples, grouped according to the culture result*.

### Detection of specific antibodies by western blot

**Figure 2:**
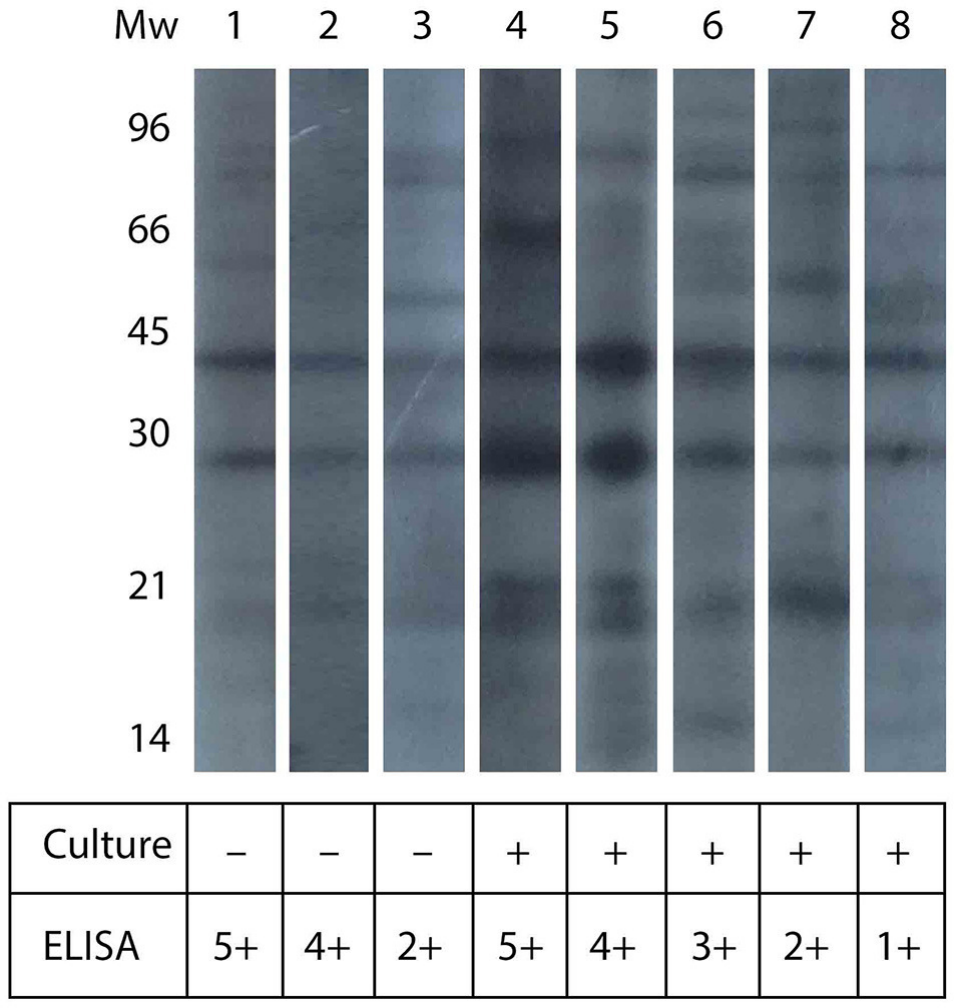
**Western blot analysis of llama serum samples**. The figure shows the western blot analysis for serum samples from culture-negative and culture-positive animals (as indicated in the table below). The figure also shows the relative intensity in ELISA signal, in arbitrary units.

Western blot analyses were conducted in serum samples that yielded the higher values by ELISA, some of them being positive for the microbiological analysis, and some being negative. Figure 2 shows the pattern of antigen recognition for three culture-negative and five culture-positive serum samples. The figure shows that almost all serum samples recognized two bands of about 30 and 45 kDa, and a set of smaller molecules of around 21 kDa. The antigen-recognition pattern did not differ significantly among culture-negative and culture-positive serum samples.

### Serological survey in animals from other regions within argentina

In order to assess whether the high prevalence of *Acinetobacter* colonization and the lack of difference in serological tests between colonized and non-colonized animals was exclusive of the Catamarca province, we studied the prevalence of reactive antibodies against *A. lwoffii* in serum samples from other provinces of Argentina. Results (summarized in Table 3) showed variable levels of antibodies among the different regions analyzed. When all samples were considered as a single group, independently of the direct culture analysis, O.D. measurements of each region behaved as a set of normally distributed values. Nevertheless, the Kruskal Wallis non-parametric test was performed, due to the different number of values in each group, and the result indicated that mean O.D. values differed between regions. Furthermore, by the Dunn's Multiple comparisons post-test it was found that the Buenos Aires region had significantly higher mean value as compared to the other regions, save Entre Ríos. This test also showed that the Catamarca region differs from Entre Ríos region.

**Table 3:** **Comparison of O.D. values from different regions within Argentina**.

**Province**	***n***	**Mean**	***S.D***.	**Min**	**Max**	**Median**	**p s-w**
Catamarca	36	0.48	0.43	0.08	2.01	0.33	<0.0001
Buenos Aires	31	1.23	0.77	0.39	2.71	0.98	<0.0001
Entre Ríos	24	0.99	0.79	0.01	2.47	0.69	<0.0001
San Luis	10	0.86	0.59	0.39	2.47	0.71	0.0004
Chubut	12	0.46	0.3	0.13	1.29	0.38	0.01
Jujuy	18	0.64	0.51	0.19	2.22	0.43	<0.0001

The analysis of outliers was performed, applying the procedure in an iterative manner, recalculating the parameters until no outliers could be detected. This strategy resulted in no outliers detected after two iterations of the robust test (Table 4).

**Table 4:** **Analysis of ELISA outliers for each region**.

	**Outliers (it. #1)**	**P (Unilateral D)**	**Outliers (it. #2)**	**P (Unilateral D)**
Catamarca	5	0.003	1	0.02
Buenos Aires	0	<0.0001		
Entre Ríos	0	<0.0001		
San Luis	1	0.5605		
Chubut	1	0.2737		
Jujuy	2	0.178		

**Figure 3:**
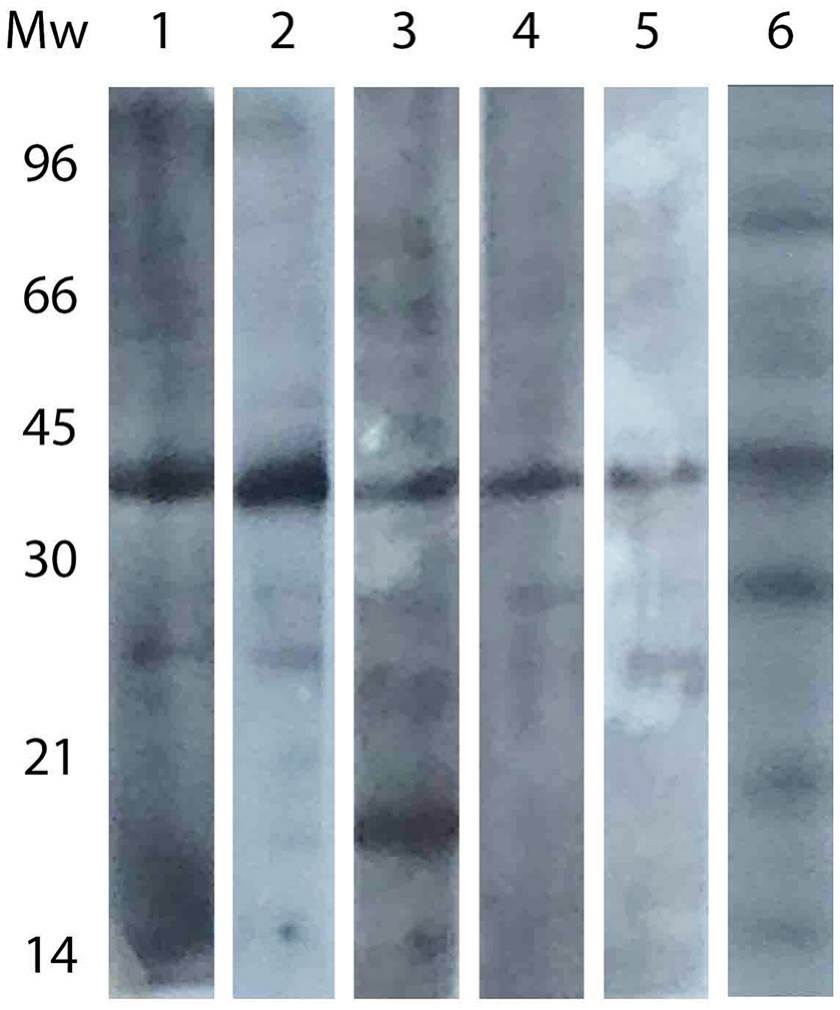
**Western blot analysis of sera from different regions**. Lanes: 1, Buenos Aires; 2, Entre Ríos; 3, Jujuy; 4, Chubut; 5, San Luis; 6, Catamarca (same strip as that presented in Figure 2).

A Western Blot assay was performed with samples belonging to different regions. As shown in Figure 3, the pattern of antigen recognition differed among regions. Except for Catamarca, the band at 30 kDa did not appear in any region, and an additional protein was recognized in one sample at a molecular weight lower than 21 kDa (sample from Jujuy). Nevertheless, no specific pattern could be assigned to neither positive nor negative samples.

## Discussion

Bacteria belonging to the genus *Acinetobacter* are short pleomorphic Gram-negative *bacillococci*. DNA hybridization techniques have allowed the description of 20 species within the genus. Identification at species level is complicated, mainly due to the high degree of similarity between individuals. Most species within the *Acinetobacter* genus are opportunistic pathogens, especially *Acinetobacter baumannii*, which is one of the major problems in infectology due to the high frequency of drug multiresistance and its wide distribution in the environment. *A. baumanii* is the cause of serious nosocomial infections, especially in intensive care units. *Acinetobacter* infection may be endemic or epidemic, with important and hard-to-limit outbreaks. It is unusual for Gram negative bacteria to live in the ambient after desiccation, but not only does this genre survives, but also remains viable for causing infection. Literature indicates that researchers Allen and Green were the first to report that airborne dissemination can be the way of transmission in outbreaks (Leardini, 2010).

The incidence of *Acinetobacter* other than *A. baumanii* was studied by others (Turton et al., 2010) and with a remarkable depth and precision by Al Atrouni et al. (2016). Although most clinical isolates were identified as *A. baumannii* (78%), authors indicated that a significant number of other species were found, particularly *A. lwoffii* (8.8%), *A. ursingii* (4%), genospecies 3 (1.7%), and *A. johnsonii* (1.7%), often associated with bacteremia. These species have been associated with serious infections, and may be considered emerging pathogens. Indeed, *A. lwoffii* represents 61% of the isolates of *Acinetobacter* spp. other than *A. baumannii*, and the available information indicates that these isolates are combined with clinical symptoms of sepsis, bacteremia, pyrexia, meningitis, post-hemorrhagic hydrocephalus, rigors, pneumonia, cellulitis, rash, ophthalmia neonatum, urinary tract infection, and abscess. Moreover, some authors (Tega et al., 2007) reported the isolation of multi drug resistance *A. lwoffii* strains in cases of catheter-related bacteremia, and bacteremia has been associated to gastroenteritis (Regalado et al., 2009).

Up to date, *Acinetobacter* non-*baumanii* had been reported in the environment, food and animals. According to literature (Al Atrouni et al., 2016), these bacteria have been isolated from cows, horses, pets, some aquatic animals and even lice collected in homeless shelters or the head of primary school pupils. In the present work we report for the first time the colonization of *L. glama* with bacteria from the *Acinetobacter* genre. The cause of infection remains unknown, but the finding is relevant given that these microorganisms might be the cause of infection among cattle, and even humans.

The serological survey conducted herein reveals that the presence of specific antibodies does not have a clear correlation with the presence of bacteria, given that the statistical analyses lead to 76.9% of false negatives (10 ELISA-negative animals out of 13 culture positive animals) and 15.4% of false positives (2 ELISA-positive animals out of 13 culture negative animals). Furthermore, O.D. mean values of the positive and negative groups is not significantly different. Furthermore, Western blot analyses revealed the absence of a consistent antigen-recognition pattern, a result also demonstrated when animals from other regions—different from Catamarca—were included in the study. Although this lack of correlation might be due to a misclassification of animals, we believe it may be the consequence of a low prevalence of specific antibodies in long-term colonized animals, or even to the presence of naturally occurring specific antibodies in animals, in the absence of *Acinetobacter*.

In order to evaluate the use of serology for clinical diagnosis in the context of respiratory pathogens of SACs, some speculations can be derived from the O.D. mean values. The presence of outliers might be indicative of true positive colonization (positive by ELISA and direct culture), although this was contradicted in Catamarca by the presence of high ELISA O.D. values corresponding to culture–negative animals. However, an assumption can be made that these animals could have been misclassified (by inadequate nasopharingeal sampling), and therefore the presence of ELISA outliers could still be an indicative of true colonized animals. If that were the case, any non-normal distribution of O.D. values might be indicative of the presence of colonized animals, especially if outliers can be detected and a normal distribution is achieved after their elimination.

Finally, animals belonging to Buenos Aires and Entre Ríos had higher O.D. values, possibly indicating the presence of other antibodies cross-reacting with *Acinetobacter*, or less probably, a wide-spread colonization by this microorganism.

## Conclusion

In the present work, colonization of *L. glama* by bacteria of the *Acinetobacter* genus was demonstrated for the first time. These bacteria had previously been reported in the environment, food and animals, but never in camelids. Serological tests conducted herein were demonstrated not to be useful to predict colonization, although some speculations can be made regarding the distribution of O.D. values. Given that these microorganisms might be the cause of infection among cattle, and even humans, this report contributes to a better understanding of the microbiota present in South American camelids. Although serology has proven not to be useful for clinical diagnosis of *Acinetobacter* in these animals, the use of nasopharingeal swabs resulted in a practical, inexpensive method for assessing the identity of bacteria colonizing the upper respiratory tract. Further studies should be conducted to analyze the origin of cross-reacting antibodies.

## Ethics statement

The study was performed under the guidance of the Ethics Committee of the Pharmacy and Biochemistry School, University of Buenos Aires, Argentina. Procedures were approved by animal owners under a written contract, which explicitly indicated that no harm would be done to any of the participant animals, and that the appropriate care would be given if any of them were affected by the procedures. Nevertheless, no animal was injured. No vulnerable populations were involved.

## Author contributions

All authors contributed to the work, during planification, experiment performance, and result discussion. ML performed most of the experiments, and AF leaded the project.

### Conflict of interest statement

The authors declare that the research was conducted in the absence of any commercial or financial relationships that could be construed as a potential conflict of interest.
